# Supplementary material for: Identification of Crown of Thorns Starfish (COTS) using Convolutional Neural Network (CNN) and attention model
Source: PLoS One. 2023 Apr 5;18(4):e0283121. doi: 10.1371/journal.pone.0283121 (PMC10075425; doi:10.1371/journal.pone.0283121)
Supplement: Attachment — Submitted filename: Response to Reviewer.docx [file pone.0283121.s001.docx]

Response to Reviewer

**Journal Requirements:**

When submitting your revision, we need you to address these additional requirements.

1. Please ensure that your manuscript meets PLOS ONE's style requirements, including those for file naming. The PLOS ONE style templates can be found at 
<https://journals.plos.org/plosone/s/file?id=wjVg/PLOSOne_formatting_sample_main_body.pdf> and 
<https://journals.plos.org/plosone/s/file?id=ba62/PLOSOne_formatting_sample_title_authors_affiliations.pdf>

**File naming revised**

2. Please note that PLOS ONE has specific guidelines on code sharing for submissions in which author-generated code underpins the findings in the manuscript. In these cases, all author-generated code must be made available without restrictions upon publication of the work. Please review our guidelines at <https://journals.plos.org/plosone/s/materials-and-software-sharing#loc-sharing-code> and ensure that your code is shared in a way that follows best practice and facilitates reproducibility and reuse.

**Codes are available upon request and may be uploaded on GitHub if required.**

3. Please ensure that you include a title page within your main document. We do appreciate that you have a title page document uploaded as a separate file, however, as per our author guidelines (<http://journals.plos.org/plosone/s/submission-guidelines#loc-title-page>) we do require this to be part of the manuscript file itself and not uploaded separately.
Could you therefore please include the title page into the beginning of your manuscript file itself, listing all authors and affiliations.

**Title page has been included**

4. Thank you for stating the following in your Competing Interests section:

"NO authors have competing interests"

Please complete your Competing Interests on the online submission form to state any Competing Interests. If you have no competing interests, please state "The authors have declared that no competing interests exist.", as detailed online in our guide for authors at <http://journals.plos.org/plosone/s/submit-now> 
 This information should be included in your cover letter; we will change the online submission form on your behalf.

**This has been added in the cover letter.**

5. In your Data Availability statement, you have not specified where the minimal data set underlying the results described in your manuscript can be found. PLOS defines a study's minimal data set as the underlying data used to reach the conclusions drawn in the manuscript and any additional data required to replicate the reported study findings in their entirety. All PLOS journals require that the minimal data set be made fully available. For more information about our data policy, please see <http://journals.plos.org/plosone/s/data-availability>.

Upon re-submitting your revised manuscript, please upload your study’s minimal underlying data set as either Supporting Information files or to a stable, public repository and include the relevant URLs, DOIs, or accession numbers within your revised cover letter. For a list of acceptable repositories, please see <http://journals.plos.org/plosone/s/data-availability#loc-recommended-repositories>. Any potentially identifying patient information must be fully anonymized.

Important: If there are ethical or legal restrictions to sharing your data publicly, please explain these restrictions in detail. Please see our guidelines for more information on what we consider unacceptable restrictions to publicly sharing data: <http://journals.plos.org/plosone/s/data-availability#loc-unacceptable-data-access-restrictions>. Note that it is not acceptable for the authors to be the sole named individuals responsible for ensuring data access.

We will update your Data Availability statement to reflect the information you provide in your cover letter.

[Note: HTML markup is below. Please do not edit.]

**We have added the links in the cover letter**

**Response to the reviewer’s Comments**

**Reviewer #1: The authors needs to update their research article as per the below mentioned comments:**

| 1. The difficulties of snorkelling and diving must be discussed by the author in order to identify COTS. | **The difficulties of snorkelling and diving was mentioned in the abstract.**  **We have added the following the the abstract:** Where strong currents result in poor image capture, damage of capturing equipment, and are of high risks. |
| --- | --- |
| 2. The author should update Fig. 2. (Improve the readability of Fig. 2) | This has been updated to a higher resolution |
| 3. Modify Fig. 3, Fig. 6. Difficult to read | This has been updated to a higher resolution |
| 4. Change Fig. 9 (Captions of Fig. 9(a) to Fig. 9(i) are not clearly visible) | This has been updated to a higher resolution |
| 6. Authors must include the full forms of YOLO4 and VGG19 in the Contribution section. | We have put the full form of YOLOV4 and VGG19 in the contribution section |
| 7. The authors have used VGG19 with CBM, which performs better than earlier models. The characteristics of the convolutional block attention module (CBAM) must be described by authors | **Section 3.5 has been updated.** |
|  |  |

Reviewer #2: The following points need to be looked upon in the manuscript.

| 1. The review of literature provides an extremely low number of papers in Table 1. Are these papers important ones for the readers with respect to the topic of research, or only these many number of papers are available in this field? | These are the only papers in this field at the time of submission. Only these authors have worked in the area of COTs and deep learning |
| --- | --- |
| 2. The datasets links must be changed to the citations and provide the links as part of reference. | The manuscript has been updated accordingly. |
| 3. The figures are not placed in the proper places in the manuscript. | Figures have been referred by the caption. Figures have been placed so as not to disrupt the format of the manuscript and have been placed following their mention in the paper. However, since images are uploaded separately from the main text, and the final Pdf is generated automatically, the generated Pdf may be different from the intended manuscript. |
| 4. The extraction of features using VGG16 and MobileNetV2 are already proposed ones. The manuscript must highlight the importance of using these techniques in the proposal. Also, justify their usage in comparison with other techniques available in this domain. | It is mentioned in Section 3.4 that :  ***Despite the promising results of deep learning, the latter has not yet been explored in the detection of COTS***  We have added the following to address this comment:  Instead of using a single Convolution layer with a big kernel size, the VGG network introduced the idea of grouping many convolution layers with smaller kernel sizes. As a result, there were fewer features at the output, and adding three RELU layers rather than one increased the number of learning instances. On the other hand, MobileNet-v2 is a convolutional neural network that is 53 layers deep, which has been trained with over one million images from the ImageNet dataset.  We have also highlighted the importance of these techniques over machine learning techniques.  We have added the following:  Previously machine learning techniques were popular in feature extraction and selection. Machine learning refers to the use of algorithms by computers to learn from data and carry out tasks automatically without explicit programming. In contrast, deep learning employs a powerful set of algorithms that are designed after the human brain. This makes it possible to process unstructured data, including text, photos, and documents. In this case, deep learning is able to extract complex features from the images. |
| 5. Table 3 indicates the performance comparison. Also provide a written explanation on why these values are obtained as such and reason of such comparative values. Justify the proposed model's uniqueness in being better than the other approaches. | Compare it with results in machine learning – COTS |
| 6. The results are not adequate and lacks the amount of work done to justify the proposal. | The section Results and Discussions has been amended. |
| 7. The discussion on future scope of work must be clearly explained. | The following has been added in the conclusion section:  The proposed system can be used in real-time for the detection and classification of COTS in the lagoons to help detect and manage COTS outbreaks. |

**Reviewer #3: Review**
Abstract: - Is there any improvement of map (mean average precision), Loss and FPS of proposed model (attention based with CNN) over VGG-19 and MobileNetV2 model? Please give improvement results in percentage.

This has been taken care of in the abstract.

Dataset:- Number of images in data sets are not enough large for approximate results. Is there any large size of data set available?

The Kaggle dataset was the largest publicly available dataset. In addition, data augmentation techniques were applied to increase the number of images in the dataset.

No any figures are available or visible like fig 1,2,3,4……etc in that manuscript. Kindly provide the all figures.

Images are uploaded separately from the main text, and the final Pdf is generated automatically, the generated Pdf may be different from the intended manuscript. Image quality has been further enhanced and re-submitted.

Proposed Model:- In that manuscript ADAM used as optimizer along with VGG-19 and MobileNetV2 . How did you optimize your attention based model (CBAM) for further enhancement of VGG19? Moreover there is no any working process and math behind the reason about proposed model (spatial attention of CBAM) so that it could be understood that how attention model focused only on the particular area of COTS even how could fed the CBAM along with CNN variant’s layer as so called optimizer/enhancement. Kindly provide the working process and math of the proposed model.

More details of the proposed model have been added with some Mathematical equations.

Results and Discussion: - In that manuscript, in results section,YoLOV4 version used to detect the starfish but why did not compare YOLOV4 results with your proposed model and other VGG19 and MobileNetV2? Wrote there in manuscript “Our algorithm has achieved an accuracy of 95%”, is that YOLOV4 accuracy? Or your proposed model accuracy? There is lot confusion.

95% was for the precision. The text has been amended accordingly.

Is YOLOV4’s result better than of your proposed model (CBAM)? Kindly provide the results of YOLO V4 and compare it with proposed model along their graphs.
In figure 10, there should have testing accuracy and loss results of all variant too. Kindly provide.

Please note that YOLO has been used for detection and CBAM for classification and Explainability

6. PLOS authors have the option to publish the peer review history of their article ([what does this mean?](https://journals.plos.org/plosone/s/editorial-and-peer-review-process#loc-peer-review-history)). If published, this will include your full peer review and any attached files.

If you choose “no”, your identity will remain anonymous but your review may still be made public.

**Do you want your identity to be public for this peer review?** For information about this choice, including consent withdrawal, please see our [Privacy Policy](https://www.plos.org/privacy-policy).

Reviewer #1: No

Reviewer #2: No

Reviewer #3: **Yes:** Manjit Jaiswal
